# Supplementary material for: Are carbon emissions trading and green financial instruments synergistic? -Comprehensive quantitative research based on content analysis
Source: PLoS One. 2024 Mar 7;19(3):e0298601. doi: 10.1371/journal.pone.0298601 (PMC10919601; doi:10.1371/journal.pone.0298601)
Supplement: S1 Data — (ZIP) [file pone.0298601.s001.zip › Data/地级市Z_F工作报告.zip!/┴╔─■/╔≥╤⌠╩╨/└┤╫╘╣1⁄2╓┌║┼í╛┬φ┐╦╩2╛▌═°í┐_2018.docx]

各位代表：

　　现在，我代表市政府向大会报告工作，请予审议，并请市政协委员提出意见。

　　一、过去五年及2017年工作回顾

　　过去五年，是沈阳振兴发展进程中极不平凡的五年。我们深入学习贯彻习近平新时代中国特色社会主义思想，全面落实新发展理念和“四个着力”“三个推进”要求，坚持稳中求进工作总基调，积极应对严峻复杂的经济形势和巨大的下行压力，努力克服政治生态遭受破坏带来的严重影响，砥砺奋进，开拓创新，推动沈阳振兴发展迈出坚实步伐。

　　政治生态持续净化修复。坚决肃清王珉及苏宏章等人流毒和辽宁拉票贿选案恶劣影响，自觉践行“讲诚信、懂规矩、守纪律”要求，挤出水分，做实数据，实现了有质量有效益发展。扎实开展群众路线教育实践活动、“三严三实”专题教育，推进“两学一做”学习教育常态化制度化，贯彻落实中央八项规定精神，政府自身建设不断加强。

　　振兴发展思路举措更加清晰。坚持从习近平总书记关于东北振兴的重要指示批示中找方向、找路径、找方法，围绕落实“五大区域发展战略”和建设“一带五基地”，绘就发展蓝图，出台战略规划，强化“项目为王”意识，弘扬“马上办”“钉钉子”精神，推进转型创新发展成为全市上下共同意志和共同行动。

　　产业结构逐步优化。制造业加快迈向中高端，高端装备制造业产值占比从16%提高到23%。金融、物流、旅游、会展等现代服务业加快发展。高产生态、高端精品、高效特色农业规模不断壮大。高新技术产品产值占比从41%提高到55%，120万吨乙烯三机、AP1000核主泵、i5智能数控系统等60余项大国重器在沈问世，“沈阳制造”为天宫、蛟龙、神州、航母、大飞机等国家重大工程作出了贡献。

　　改革开放不断深化。我市成为全国8个全面创新改革试验区之一，自主创新示范区、自贸试验区、中德装备园、中国制造2025试点示范城市、大数据综合试验区等“国字号”改革试点深入实施。引进来、走出去取得新成效，在沈投资世界500强达到94户，国际友城达到20个，开通沈满欧、沈连欧两条中欧班列，全市累计实际利用外资109.7亿美元、进出口总额691亿美元。

　　城乡面貌显著改善。确立“东山西水、一河两岸、一主三副”的城市发展新格局，新型城镇化和城乡一体化稳步推进，城镇化率达到80.5%。综合立体交通体系基本形成，新建地铁工程94.7公里，桃仙机场T3航站楼、沈阳南站等重大交通枢纽投入使用，城市快速路、农村公路大幅增加。智慧城市建设全面展开，经济社会加快步入“互联网+”时代。

　　生态环境明显好转。认真落实主体功能区制度，划定城市开发边界、生态控制线和基本农田保护线。退耕还林还湿还草还湖政策体系不断完善，森林覆盖率持续提高。空气质量优良天数不断增加，土壤环境保持稳定，水环境得到较大改善。涉农区县全部成为国家级生态县。我市成为国家循环经济示范城市。

　　人民生活水平稳步提升。城乡居民人均可支配收入年均增长6%以上，城镇登记失业率控制在3.4%以内。多层次社会保障体系基本形成，群众住房条件不断改善。各级各类教育协调发展，医疗卫生服务能力显著增强，盛京大剧院、沈阳艺术大厦等文化设施投入使用，第十二届全运会等重大赛事在沈举办。我市成功蝉联全国文明城市。

　　五年来，我们认真贯彻中央新一轮振兴战略，全面落实中央7号文件、国务院28号文件和62号文件要求，主动适应把握引领新常态，以供给侧结构性改革为主线，统筹推进稳增长、促改革、调结构、惠民生、防风险各项工作，克服“三期叠加”影响，摆脱惯性思维，突破路径依赖，收获了接力奋进的可喜成果!

　　刚刚过去的2017年，是本届政府收官之年，是扭转经济下行态势、推动振兴发展任务极其繁重的一年。我们把学习贯彻党的十九大精神作为首要政治任务，凝心聚力，负重前行，克难攻坚，推动沈阳转型创新发展取得新成效。

　　预计地区生产总值完成5870亿元，比上年增长3.5%；一般公共预算收入656.2亿元，增长5.7%，税收收入占比达到81.8%；城镇居民人均可支配收入41359元，增长6.1%；农村居民人均可支配收入15461元，增长7.5%。

　　经济社会发展呈现出一些新特点。一是经济运行企稳向好。地区生产总值结束6个季度负增长，规模以上工业增加值结束32个月负增长。工业用电量连续11个月增长，货运总量连续21个月增长，PMI连续11个月运行在荣枯线以上。二是一批基础性关键性改革全面展开。国资国企改革、“多规合一”改革取得重要成果，科技体制、投融资体制、放管服、商事制度等改革不断深化，全面创新改革形成了23项可复制推广经验，我市打造国际化营商环境入选2017年度全国十大优秀改革案例。三是一系列制度性安排启动实施。围绕振兴实体经济、推动转型创新发展、提升城市品质、保障改善民生、加强干部队伍建设等方面，市委市政府出台了17个政策性制度性文件，为促进振兴发展提供了有力保障。四是一些积久积重的难点问题得到解决。建成区基本消灭黑臭水体，630处燃气管网占压问题得到整治，盛京输变电工程重新选址建设，祝家污泥启动无害化处置，西部污水处理厂二期投入运行，大辛、西部、老虎冲垃圾焚烧发电项目开工建设，环保部挂牌督办的炼焦煤气厂停产关闭。五是干部群众精神状态昂扬向上。随着政治生态的净化修复，干事创业环境焕然一新，干部群众焕发出强烈的奉献精神、担当精神、攻坚精神、创新精神。一年来，沈阳发生了许多变化，这些变化是由内而外的，从理念、思路、态度，逐步向方法、模式、作风等层面展开；这些变化是深刻的、是持久的，是沈阳加快振兴发展的信心所在、力量所在!

　　一年来，我们主要做了以下工作：

　　(一)推动国际化营商环境不断改善。围绕七大环境建设，坚持问题导向，突出专项整治，对照世界银行标准，我市营商环境预计达到前70位经济体水平。

　　开展精准对标。聘请第三方机构，针对企业全生命周期各项指标做评估、找差距。组建市营商办和营商环境监督局，强化明察暗访、驻局督办，累计解决群众诉求14.2万件、问责825人次，形成71条可复制推广的工作举措。营商环境“企业评、群众议”认同度达到82.2%。

　　实施专项整治。整治政务窗口服务差问题，投诉量下降90%。整治乱检查乱收费乱罚款问题，实施行政执法检查计划管理，涉企行政处罚案件减少91%。整治垄断性行业服务质量问题，制定供水、供气、供热等服务规范，加快放开中介服务市场。整治政府和企业失信问题，落实政府还款计划，建立失信企业“黑名单”。整治企业跨区迁移难问题，基本消除了行政干预等壁垒。

　　深化放管服改革。取消下放调整职权218项，推行“多证合一”“最多跑一次”、延时服务等制度，企业登记、投资审批、消防审核、获得电力等手续办理更加便利，新登记市场主体16.9万户，增长62.4%。“多规合一”、营商环境、环保110、公共安全、商事服务、智慧城管等平台投入运行，促进了工作流程再造和行政效能提升。

　　(二)推动全面深化改革取得新进展。以全面创新改革试验为统领，深化重点领域和关键环节改革，一些制约发展的体制机制障碍逐步破解。

　　供给侧结构性改革成效明显。新增国际产能合作项目40个，全面取缔地条钢生产企业，处置“僵尸企业”84户。出台“13+9”房地产新政，强化分类施策，开展住房租赁试点，稳房价、去库存取得实效。坚持市场化法治化推动企业债务风险处置，超额完成政府化债任务。着力降低实体经济成本，为企业减负73亿元。服务业、农业、教育、资源环境等领域供给侧结构性改革深入开展。

　　国资国企改革实现重大突破。国企改革和国资平台建设联动推进、精干主体和解决历史遗留问题同步推进、“一企一策”和整体改革政策措施配套推进、加强国企党建和强化国资监管协同推进。市领导一对一、点对点推进重点国企改革，机床集团综合改革成为国家试点，沈鼓集团等6户重点企业改革取得阶段性成果，市属国企整体减亏15亿元。“3+1+N”国有资本运营架构初步形成，金控、城投、产投、资产管理公司等国资平台投入运营。出台国资监管清单，市属经营性国有资产实现集中统一监管。“双向进入、交叉任职”领导体制不断完善，市场化选聘经理层等制度启动实施。62户国企“三供一业”分离移交。

　　“多规合一”改革取得重要成果。坚持统筹规划、规划统筹，强化战略引领和规划管控，促进了项目主导向规划主导转变。沈阳城市总体规划获国务院批复，启动新一版总体规划编制改革试点，一张蓝图实现规划区全覆盖，“多规合一”平台市、区(县市)两级全面贯通。

　　城市管理体制不断完善。深化城市管理执法体制改革，整合市政公用、市容环卫、园林绿化等执法职能。完成土地管理体制转轨，实现规划、储备、出让、分配、存量盘活“五个统筹”。加快不动产登记制度改革，实现土地证、房产证“两证合一”。全面启动供排污一体化大水务改革，着力破解“多龙治水”难题。

　　(三)推动新旧发展动能加快转换。坚持“项目为王”，强化创新驱动，积极培育新的经济增长点。

　　项目建设扎实推进。实施重大项目领导全程包保，重点项目督查督办，切实解决规划、征拆、配套等受阻问题，推进制造业、服务业、环保、交通、能源等9大工程包亿元以上项目631个，完成投资1280亿元。米其林轮胎扩产、盛京金融广场等项目加快建设，华晨宝马新大东工厂、东北世贸广场等项目投产运营。

　　创新创业日益活跃。构建“1+4”科技创新政策体系，启动实施“1123工程”，极限尺寸纳米金属材料等490项关键技术实现突破，高新技术企业增加292家，总量跃居东北首位。材料科学国家研究中心、国家机器人创新中心等重大创新平台在沈布局，沈阳科技条件平台投入使用，众创空间、科技孵化器达到106家。建立市长与大学校长、科研院所长联席会议制度，全面复制中关村“6+4”政策，科技成果使用权、处置权、收益权改革不断深化，东北科技大市场投入运营，全市技术合同成交额增长16.4%。加大招才引智力度，出台24条人才新政，扎实开展“三引三回”活动，人才回流趋势明显，呈现出“春暖燕归来”的积极变化！

　　军民融合不断深化。产业联盟单位达到117家，增材制造、旋翼无人机等一批前沿技术实现军转民，民参军企业突破100家。皇姑、浑南、沈北等园区加快建设，明日宇航、和世泰等项目顺利推进。航天十二院军民融合中心、航天科技软件测评中心、国家保密科技测评中心落户沈阳，中国军民融合门户网上线运行。

　　民营经济稳步发展。新增个转企2601户、规上限上企业727户。设立1亿元小微企业流动性债权基金，应急转贷资金扩大到10亿元，小微双创资金投入提高51%，缓解了企业融资难问题。建成8个省级中小企业公共服务平台、5个中小微企业创业(辅导)基地，新增40项省定“专精特新”产品。社会资本加快进入金融、医疗、健康等领域，辽宁振兴银行获批开业，辽宁民营投资集团注册成立，沈阳积水潭医院、东北国际医院投入运营。

　　(四)推动产业转型升级迈出新步伐。坚持转方式、调结构，积极培育增量、做优存量，我市成为国家首批转型升级示范区。

　　战略性新兴产业快速发展。机器人、航空、新一代信息技术、生物制药及高性能医疗器械等产业产值分别增长28.6%、12.5%、8.2%、11.6%。龙头企业带动作用和创新平台溢出效应不断增强，新松智慧产业园投入运营，泰克南P2006T通用飞机实现量产，壮龙多旋翼飞行器等产品达到国际领先水平，国内首个12英寸半导体薄膜设备生产基地投产，国家眼基因库落户沈阳，华晨宝马新能源电池PACK工厂竣工投产。国家大数据综合试验区聚集企业130余家，建成东软云、华为云、浪潮云等多个云平台，沈阳加速向云城市迈进。

　　传统制造业加快升级。出台和落实产业转型、智能升级三年行动计划，实施149个智能升级、170个工业技改、21个工业节能项目，机床集团、沈鼓集团、特变沈变、北方重工等重点企业的高端产品占比均超过50%，利源轨道地铁车辆出口印度尼西亚。东软、中科院沈阳自动化所入选工信部首批智能制造系统解决方案供应商，东北首个“互联网+检验检测认证”公共服务平台上线运行。

　　服务业加快发展。全市新增金融及金融服务机构44家，15家企业“新三板”挂牌，84家企业辽股交挂牌。国家级旅游业改革创新先行区启动建设，熊猫馆、七星海世界等项目建成开放，旅游业总收入增长13.1%。举办各类展会405项，交易额增长10.1%。沈阳铁路综合货场等物流园区投入运营，浑南电商示范基地进入全国三甲，建成小笨鸟、聚贸、五爱等跨境电商平台，全市限上企业网上商品零售额增长37.4%。国际软件园跻身全国最佳科技服务园区十强。开展“送温暖、送服务”活动，促进实体零售业逐步回暖。

　　农业结构加快调整。调减玉米种植18.3万亩，建设高标准农田19.3万亩，设施农业达到27.6万亩，高效特色作物达到176万亩。农村一二三产业融合发展，建设6个亿元以上农产品深加工项目、64个休闲农业项目，培育新型经营主体947家、农村电商200家，创建14个省级以上农产品品牌，法库县成为国家级畜牧业绿色发展示范县。土地确权登记基本完成，国有林场改革通过省级验收。

　　(五)推动对外开放迈上新台阶。坚持引进来和走出去相结合，开放的深度和广度进一步拓展。

　　开放平台建设取得新突破。自贸试验区推出200条政策清单，国际贸易“单一窗口”上线运行，开展“证照分离”和集报集缴通关改革，开通审批绿色通道，注册企业达到1.4万户。中德装备园成为国家智能制造试点示范园区，中德开、中德发正式运营，落地项目171个。沈阳港重组工作有序推进。口岸功能不断完善，进口商品指定口岸增加到4类。

　　对外经贸合作不断扩大。举办正和岛全球创新大集、中国沈阳投资与贸易对接会等经贸洽谈活动，组织企业参加中国—东盟博览会、德国汉诺威工业博览会等会展活动，全市实际利用外资增长24.1%，实际到位内资增长12.8%。推行进口直通、出口直放政策，全市进出口总额增长15.9%。引导企业入驻境外合作园区，全市对外投资实现3.6亿美元。

　　区域合作呈现新局面。积极落实沈阳经济区发展战略，与鞍山、抚顺、本溪、辽阳签署共同行动计划。建立京沈对口合作机制，举办高层会商、投资洽谈、展会推介、人才交流等活动百余项，推进京东商城、联东U谷、清华启迪、北京二商等合作项目139个。同时，与江苏、厦门、深圳、延安等省市达成合作项目107个。

　　(六)推动城市治理取得明显成效。坚持精准规划、精致建设、精细管理，实施“治改提创”工程，促进城市内涵式发展。

　　加快基础设施建设。地铁2号线北延线投入试运行，4、9、10号线建设顺利实施。南北快速干道、北一路高架桥等快速路工程竣工通车，王家湾、云龙湖大桥以及五爱、富民互通立交投入使用，改造交通堵点18处，缓解了城市交通压力。实施“一河两岸”提升工程，郎朗钢琴广场、云飏阁、国际马拉松赛道等市民休闲设施建成开放。我市老城区第一条综合管廊投入使用。

　　着力补齐城市运行短板。统筹实施老旧管网改造，完成供水内网1048公里、燃气管网197公里、供热管网250.1公里。实施26项电力工程，解决部分地区电网严重过载、用电紧张问题。老旧住宅电梯维修更新计划全面完成，城市排水防涝三年行动计划启动实施。以背街小巷、老旧小区、农贸市场、“八小”行业为重点开展集中整治，市容环境进一步改善。公交都市加快建设，公交分担率和乘车舒适度不断提高。

　　大力改善生态环境。中央环保督察交办问题整改取得显著成效。实施科学治霾、精准治霾、协同治霾、铁腕治霾，建立监测监控网络、污染源解析系统和应急响应体系，加强挥发性有机物、散煤、扬尘、秸秆焚烧管控治理，拆除燃煤锅炉1115台，淘汰黄标车及老旧车3.14万辆，完成型煤替代6197户，空气质量优良天数达到256天，各项指标均创下2013年国家新标准实施以来的最好水平。建立河长制，启动“一河一策”治理管护试点。强化垃圾减量化、资源化、无害化处理，大辛垃圾场渗沥液处置项目投入试运行，全市30个垃圾村全部完成整改。加大节能减排力度，我市成为国家低碳试点城市。

　　(七)推动人民生活持续改善。坚持以人民为中心的发展思想，财政支出用于民生比重达到84.3%，人民群众获得感进一步增强。

　　加大就业和社会保障力度。城镇新增就业9.7万人，登记失业率3.12%。养老金按时足额发放，城市、农村低保标准分别提高11.4%和17.3%，医保实现全国异地就医直接结算。新建20个区域性居家养老服务中心、30个社区日间照料站，新增养老床位4184张。针对低保人员、特困供养人员、孤儿和建档立卡贫困人口的医疗、采暖等专项救助实现全覆盖，落实了困境儿童、留守儿童帮扶措施。完成棚户区改造1.2万套，实施农村危房改造805户。脱贫攻坚成效显著，贫困村全部销号，康平县甩掉了贫困县帽子。

　　加快发展社会事业。新增公办幼儿园学位3500个，改造农村义务教育薄弱学校100所，弹性离校制度荣获全国教育改革创新特别奖，高中教育优质化建设、职业教育双元制改革、市属高校转型发展取得新成果。城市公立医院综合改革稳步实施，医联体实现社区卫生服务中心全覆盖。完成20万户“问计于民”食品安全问题调查，食品药品监管得到加强。实施沈阳故宫、帅府舞厅等文化遗产保护修缮和周边环境整治，市少儿图书馆新馆一期、市老年人大学等场馆设施投入使用。沈阳国际马拉松、首届市民运动会等体育赛事成功举办。

　　推进社会治理创新。坚持共建共治共享，成立协商自治组织3000余个，新登记社会组织375个，社会治理重心加快向基层下沉。深化信访制度改革，完善专项资金体系，“融冰行动”百日攻坚成效明显，十九大安保维稳实现“六个为零”。平安沈阳建设扎实推进，安全生产改革发展、警务改革和公安基层基础建设不断加强，防范各类风险隐患能力得到提升，社会大局保持和谐稳定。

　　一年来，我们自觉接受市人大及其常委会法律监督、工作监督和市政协民主监督，提请市人大常委会审议3件法规草案，发布3件政府规章， 664件人大代表建议和568件政协提案全部办结。国家安全、国防动员、人防和民兵预备役建设进一步加强，保密、司法、统计、审计、民族、宗教、侨务、邮政、广播电视、新闻出版、气象、防震减灾、档案、修志、文史、仲裁、参事、计生、红十字会和慈善事业等工作都取得了新成绩。

　　各位代表，回顾过去的工作，我们深深感到，推动沈阳振兴发展，必须坚持正确的政治方向，高举习近平新时代中国特色社会主义思想伟大旗帜，自觉同以习近平同志为核心的党中央保持高度一致，不折不扣落实党中央的决策部署。必须坚持问题导向，聚焦企业和群众关切，勇于动真碰硬，积极化解矛盾、解决问题。必须坚持精准发力，抓住转型创新发展的关键环节，步步为营、踏石留印，增强政策措施的针对性、有效性。必须坚持真抓实干，强化担当意识，弘扬奉献精神，事事“马上办”，人人“钉钉子”，严细实快抓好落实。

　　回顾过去的工作，成绩来之不易，经验弥足珍贵。这是党中央国务院亲切关怀、省委省政府和市委坚强领导的结果，是市人大及其常委会、市政协和社会各界有效监督和大力支持的结果，是全市人民共同奋斗的结果。在此，我谨代表市政府，向全市人民，向人大代表、政协委员、各民主党派、各人民团体、社会各界人士以及离退休老同志，向驻沈人民解放军、武警部队官兵和中省直单位，向所有支持沈阳振兴发展的港澳台同胞、海外侨胞和国际友人，表示衷心的感谢！

　　各位代表，在总结成绩的同时，我们也清醒地看到，全市经济社会发展还存在诸多不平衡不充分问题。一是经济企稳向好的基础还不够牢固，地区生产总值、固定资产投资、社会消费品零售总额增速低于预期，一些传统行业面临很多困难，重大项目储备不足，产业园区主阵地作用发挥不充分，防范风险工作需要加强。二是结构调整成效不够明显，部分国家级改革试点力度需要加大，科技创新活力亟待提升，新经济、新业态规模偏小，民营经济不活跃，城乡发展不平衡。三是民生领域还有不少短板，优质的教育、医疗、健康、养老等服务供给不足，交通拥堵、环境污染、城乡接合部及背街小巷脏乱差等“城市病”仍很突出。四是政府作用发挥还不充分，该管的还没有管住管好，该放的还没有放开放活，企业和群众对营商环境改善的获得感还不强。五是工作作风还亟待转变，个别干部仍然离矛盾远、离群众远、离企业远、离项目远，不想为、不敢为、不勤为、不善为的现象依然存在。对于这些问题，我们必须加快解决。

　　二、今后五年的总体思路和主要任务

　　当前，沈阳仍处在滚石上山、爬坡过坎的关键期和攻坚期，也处于大有可为、大有作为的机遇期和窗口期，我们要在市委的正确领导下，认真落实《关于高举习近平新时代中国特色社会主义思想伟大旗帜深入学习贯彻党的十九大精神加快沈阳振兴发展的实施意见》，聚焦建设东北亚国际化中心城市、科技创新中心、先进装备智能制造中心、高品质公共服务中心的宏伟目标，全力推动高质量发展，促进质量变革、效率变革、动力变革，全面推动各项工作提速提质提效，在落实“五大区域发展战略”和推进“一带五基地”建设中干在实处、走在前列，让沈阳在新一轮振兴发展中一展雄风、大展雄风、尽展雄风！

　　(一)坚持创新发展，增强发展动力。加快科技创新，完善技术创新体系，布局重大创新平台，突出人才优先、政策引导、金融支撑、协同创新，在国家创新体系中保持战略地位。加快体制机制创新，持续深化国资国企改革，国有资本运营效益明显提高，国有企业竞争力显著增强，厂办大集体等历史遗留问题彻底解决。全面活跃民营经济，培育一批主业突出、核心竞争力强的创新型企业，把沈阳建成民营经济蓬勃发展的沃土。持续深化放管服改革，加快政府职能转变，努力建设人民满意的服务型政府。

　　(二)坚持协调发展，推进结构优化。促进产业协调发展，全面建成12条千亿产业链，创建中国制造2025国家级示范区，培育世界级先进制造业集群。促进空间协调发展，同步提升主城与副城、新城与老城的功能和品质，加快城市重点片区改造，补齐县域经济短板。促进城乡协调发展，深入实施乡村振兴战略，推动农业全面升级、农村全面进步、农民全面发展。促进区域协调发展，提升沈阳自身发展能级，带动沈阳经济区联合聚合融合，打造新型工业化示范区和具有较强竞争力、影响力的城市群。

　　(三)坚持绿色发展，建设美丽沈阳。构建绿色生产方式，推行绿色制造，壮大节能环保、清洁生产、清洁能源等绿色产业。倡导绿色生活方式，实施生活垃圾强制分类管理，开展全民节能减排行动。树立全域大生态理念，落实主体功能区战略和生态控制线管理制度，建立健全市场化、多元化生态补偿机制，加强生态整体保护、系统修复、综合治理，持续推进蓝天、碧水、青山、净土工程，坚决打赢污染防治攻坚战，坚决防止“黑色增长”，让沈阳蓝天常在、有水皆清！

　　(四)坚持开放发展，构建全面开放新格局。积极对接国际规则，引入国际标准，汇聚国际要素，完善开放型经济新体制，持之以恒建设国际化营商环境。主动融入“一带一路”，引导产能出海、境外投资、跨国并购，建设“一带一路”先行区。发挥自贸试验区制度创新优势，促进贸易投资自由化便利化，努力向自由贸易港迈进。加快沈阳港建设，创建国家临空经济示范区。拓展国际交流，加强对外联络，争取更多跨国公司、国际机构、国际展会落户沈阳。

　　(五)坚持共享发展，建设幸福沈阳。提高就业和社会保障水平，促进城乡居民收入与经济同步增长。提高教育现代化水平，促进学前教育普惠、义务教育均衡、高中教育优质、职业教育多元化市场化、高等教育转型发展。深入落实健康中国战略，建成国家卫生城市、国家食品安全示范城市、国家健康城市。积极应对老龄化问题，不断提高养老服务水平。基本完成棚户区改造，加快建立多主体供给、多渠道保障、租购并举的住房制度。大力发展竞技体育和全民健身，培育体育品牌，建设体育名城。全面提升文化品质，以文铸魂、以文化人、以文旺城、以文兴业，建设文化名城。

　　各位代表，民之所望、施政所向。我们要不忘初心、牢记使命，自强不息、埋头苦干，一件事情接着一件事情办，一年接着一年干，努力把人民群众对美好生活的向往变成现实，把沈阳建设成为具有国际品质和辽沈文化标识、宜居宜业宜游的国际化、现代化大都市，奋力谱写新时代振兴发展的沈阳篇章！

　　三、2018年重点工作安排

　　今年是贯彻党的十九大精神的开局之年，是决胜全面建成小康社会、实施“十三五”规划承上启下的关键之年，也是加快推动沈阳转型创新发展的大干实干之年。政府工作总体要求是：认真贯彻党的十九大精神，以习近平新时代中国特色社会主义思想为指导，深刻领会习近平总书记“1·5”重要讲话精神，全面落实新发展理念和“四个着力”“三个推进”要求，坚持稳中求进工作总基调，按照高质量发展要求，认真落实中央经济工作会议精神和省委、省政府及市委部署，以供给侧结构性改革为主线，围绕“四个中心”建设，突出做好加快转型升级、深化改革创新、提升城市品质、保障改善民生等各项工作，打好防范化解重大风险、精准脱贫、污染防治三大攻坚战，推动振兴发展实现大跨越！

　　今年经济社会发展主要预期目标是：地区生产总值增长7.5%左右，一般公共预算收入增长6%，固定资产投资增长10%，社会消费品零售总额增长8%；研发经费占地区生产总值比重达到2.8%，高新技术产品产值占规模以上工业比重达到57%；城乡居民人均可支配收入与经济增长同步，城镇登记失业率控制在3.7%以内；万元地区生产总值能耗下降3.6%。

　　根据目前的测算，今年全市三次产业增加值分别增长3.2%、6.5%和5%，投资、消费、出口分别增长6%、6%和11%，可以支撑地区生产总值增长6%。考虑到要与全面建成小康社会目标相衔接，与全面振兴目标相衔接，我们自加压力，将全年经济增长目标确定为7.5%左右，实现发展质量、效益和速度相统一。

　　今年重点抓好以下几方面工作：

　　(一)持续加强国际化营商环境建设。坚持问题切入、标本兼治、综合施策，营商环境排名比去年再提高10位。

　　深化问题专项整治。继续开展政府服务窗口质量、乱检查乱收费乱罚款、垄断性服务行业、政府和企业失信专项整治，将社会反映强烈的中介机构服务和政策落实“最后一公里”问题纳入整治范围。推动整治成果形成长效机制，持续开展“企业评、群众议”活动，让“不为不办找理由、只为办好想办法”成为自觉行动，使“办事依法依规、办事不用求人、办事便捷高效”成为沈阳这座城市的鲜明标签！

　　加快改善政府服务。深化放管服改革，做到放而有管、过槛即入，先批后审、有偏则纠，全程监督、跟踪服务。提高项目审批效能，推行承诺制审批和容缺受理机制，实行重大项目领办代办、建设工程“联合审图”“多测合一”。深化商事制度改革，全面推广“多证合一”，推行企业全程网上注册登记。加强政务服务标准化建设，完善“互联网+政务服务”，扩大“一窗制”服务、延时服务范围，使沈阳成为全国行政效能最高的城市之一。

　　加快改善环境条件。着力提高城市窗口形象，改善机场、火车站、汽车站、旅游景区等公共区域环境质量，进一步规范出租车等行业运营秩序。着力加强公共服务供给，积极吸引社会资本完善教育、医疗、文化、体育等服务设施，建设国际学校、国际医院、国际社区，不断提高公共服务品质。

　　加快改善要素资源配置。努力为企业获得资金、引进技术、提高管理水平创造条件、搭建平台。充分发挥政府性基金引导扶持作用，带动社会资本参与投融资。广泛开展科技成果对接、产学研对接，推动大型实验室、科学仪器、检验检测等设备开放共享。设立政府精益管理奖项，开展“标准化+”行动，弘扬工匠精神，鼓励企业提品质、创品牌。

　　(二)促进经济平稳健康发展。强化“项目为王”意识，夯实基础、增强后劲、防范风险，推动经济高质量发展。

　　狠抓投资和项目建设。紧紧把项目抓在手上，扩大数量、提高质量、加快投资进度，全年推进投资超亿元项目1000个以上，确保华晨宝马X3、米其林轮胎扩产等续建项目6月底前全部复工，华晨雷诺整车、恒大华谊艺术中心等新建项目9月底前全部开工。深化投融资体制改革，规范政府投资，引入社会资本，加快实施快速路网、海绵城市、自贸试验区基础设施等PPP项目。开展“项目推进、企业帮扶”行动，着力解决项目受阻问题。盯住目标企业、强化精准招商，全年实际利用外资、引进内资增长15%以上。

　　努力培育消费增长点。完善消费促进政策，优化消费市场环境，开展常态化消费促进活动。扩大旅游消费，发展全域旅游，培育文化、工业、乡村等旅游品牌，推动盛京皇城创建5A级旅游景区。活跃体育消费，丰富体育赛事活动，挖掘冰雪运动资源潜力，发展体育服务业。培育健康养老消费，扩大医养结合服务供给。鼓励新能源汽车消费，出台推广应用政策。加快内贸流通业改革创新，促进商贸企业线上线下融合发展，支持“老字号”创新创业。改造提升传统商圈，盘活存量商业资源，加快向生活体验综合型业态升级。

　　积极扩大出口。制定外贸发展政策，努力建设贸易强市。抓好重点市场，巩固美、日、欧等传统市场，加快开拓“一带一路”等新兴市场。抓好重点企业，推动华晨、同方、东药等50家大户扩大出口，形成一批有牵动力的出口基地。抓好重点产品，扩大成套装备、汽车、电子等产品出口规模。大力发展跨境电商平台，创建中国(沈阳)跨境电商综合试验区。全年外贸出口增长13%以上。

　　强化风险防控。建立健全地方金融监管体系，构建金融风险监控系统，提升主动发现、提前预警能力。积极防范化解非法集资风险，整顿规范金融市场秩序。坚持市场化法治化原则，稳妥审慎处置国有企业债务风险、流动性风险、上市公司退市风险。坚持“房子是用来住的，不是用来炒的”定位，保持调控政策的持续性稳定性，促进房地产市场平稳健康发展。

　　(三)深化供给侧结构性改革。坚持质量第一、效益优先，全面抓好“破立降”工作，进一步提振实体经济，着力构建现代产业新体系。

　　全力创建中国制造2025示范区。制定实施示范区创建方案，设立中国制造2025产业基金，积极对接中国制造2025五大工程。重点推动互联网、大数据、物联网、人工智能与传统产业深度融合，推广智能制造新模式，实施100个智能化生产线升级项目，建设10家示范性智能工厂，带动制造业向智能化生产、个性化定制、网络化协同、服务型制造加快升级。积极构建优势产业生态体系，推进机器人智能城、东方机器人谷等重大项目建设，打造机器人产业生态圈；依托浑南、法库、沈北等航空产业园区，壮大飞机大部件、通航产业集群；放大IC装备制造优势，延伸IC设计、材料、封装、检测产业链；启动建设东软数字医疗和国际医疗产业园、眼精准医疗小镇，构建大健康产业链。大力发展未来产业，推动现有汽车企业向电动化、智能化转型，引入知名新能源整车及配套企业，在新能源汽车产业领域实现突破；培育大数据龙头企业，开展数据增值增益加工交易，建设大数据产业集群；发展机器学习、计算机视觉、自然语言处理等人工智能技术，积极培育智能服务机器人、智能无人机、智慧医疗、可穿戴设备等人工智能产业。

　　加快发展现代服务业。推动金融商贸开发区晋升国家级，促进私募基金、融资租赁等新型金融业态集聚发展；规范发展区域股权市场，集中推动企业股份制改革，加强主板上市及“新三板”挂牌企业的动态储备。大力发展冷链物流、快递物流等物流业态，培育跨境电商、农村电商、服务业电商等电商形态。繁荣会展经济，建设以国际会展中心、新世界博览馆、工业展览馆为支撑的会展产业带，办好国际多功能材料与结构大会、国际机器人大会、国际航空博览会、制博会等大型会展活动。

　　全面活跃民营经济。出台针对性政策措施，强化政策宣传，坚决破除各种歧视性限制和隐性障碍。加强融资服务，组织银企对接，用好中小企业融资扶持资金和小微企业流动性债权基金，推行应收账款、股权、知识产权等质押融资，扩大应急转贷资金规模，启动助保贷业务。加强中小企业人才、用工服务，扩大职业教育紧缺专业办学规模。加强创新服务，培育“专精特新”中小企业，推动个转企、小升规、规转股、股上市，促进中小企业集群式发展。加强市场服务，开展产需对接、工商对接、农超对接等活动，办好2018APEC中小企业技展会。严格执行产权保护制度，落实好新修订的中小企业促进法。积极构建亲清新型政商关系，畅通政府与企业联系渠道，保护和激发各类市场主体活力。

　　做强做大县域经济。实施县域经济三年攻坚计划。突出抓好县域工业，推动产业园区改革创新、提质增效，努力形成主导产业清晰、配套体系完备、上下游聚合的特色产业集群。发挥县城和中心镇的带动作用，培育金融、物流、旅游、健康、养老等服务业态，建设县域服务业集聚区。拓展新型城镇化空间，规划建设8个特色小镇。县域主要经济指标增速要达到全市平均水平。

　　继续降低实体经济成本。落实好各项降本减负政策，进一步降低税费成本，完善税收优惠清单、涉企收费清单，挖掘行政事业性收费、政府性基金降费空间。进一步降低用能成本，扩大直购电交易覆盖企业范围。进一步降低物流成本，积极开展无车承运人试点，加快解决物流管理费用高、物流企业小散乱、物流园区功能不全等问题。

　　(四)实施乡村振兴战略。制定乡村振兴战略规划，健全城乡融合发展体制机制，促进农业农村优先发展。

　　持续推进农村改革。加快承包地“三权分置”改革，建立市县乡三级产权交易体系，扩大土地流转规模。深入推进集体产权制度改革，争取50%的村集体完成清产核资，启动集体经营性资产股份合作改革试点，壮大集体经济。创新金融支农模式，扩大土地承包经营权抵押贷款试点，建立农村征信风险补偿机制和农业信用担保平台，引导工商资本下乡，推动涉农区县农信社转制农商行。加快供销社改革和集体林权改革。

　　深化农业供给侧结构性改革。大力发展“三高型”农业，建设400万亩粮食、蔬菜、特色作物生产核心区，推动畜牧、水产养殖规模化生态化发展，持续培育寒富苹果、沈阳大米等十大农业品牌。推广“互联网+现代农业”，扩大设施农业规模，农业综合机械化率达到85%以上。创建辽中、康平两个国家级农产品质量安全县。促进一二三产业融合发展，重点建设6个农产品加工园区，打造10个休闲农业特色村，培育新型经营主体200家，发展农村电商500家。

　　加快建设宜居乡村。大力改善农村环境，建好管好护好运营好农村路，整修农村公路500公里，改造村内道路及边沟600公里，完成农村改厕2.7万户。提高农村光纤网络、4G无线网络覆盖质量。开展化肥农药使用量零增长行动，促进畜禽养殖废弃物无害化处理、资源化利用，新建改造6.86万人安全饮水设施，改造农村危房341户，建设12个宜居示范村、10个城乡融合典范村。支持康平县建设城乡融合发展示范区。加快改善农村基本公共服务，全面完成农村义务教育薄弱学校改造，建立健全农村三级医疗卫生服务网络，持续缩小城乡低保差距。

　　加强和创新乡村治理。坚持自治法治德治相结合，完善党委领导、政府负责、社会协同、公众参与、法治保障的现代乡村社会治理机制。严查“微腐败”，严打“村霸”和宗族恶势力，建设平安乡村。繁荣乡村文化，新建98处农村社区文体广场，厚植文明乡风、良好家风、淳朴民风，弘扬社会主义核心价值观。强化乡村振兴人才支撑，着力培育一批农村创业创新带头人，造就一支懂农业、爱农村、爱农民的“三农”工作队伍。

　　巩固精准脱贫成果。坚持质量优先，“摘帽”不摘政策，确保扶贫投入不减、扶贫队伍不撤。开展建档立卡贫困人口“回头看”，坚持扶贫与扶志、扶智相结合，加大贫困人口技能培训力度，激发脱贫内生动力。推广“村集体+合作社+贫困户”等产业扶贫模式，促进贫困人口稳定增收，继续抓好专项扶贫、行业扶贫、社会扶贫，坚决打赢精准脱贫攻坚战。

　　(五)加快推进创新驱动发展。以自主创新示范区建设为抓手，集聚创新资源，活跃创新要素，激发主体活力，加快建设东北亚科技创新中心。

　　不断提高创新能力。持续推进“1123工程”，支持企业加大研发投入，在机器人与智能制造、新材料、高端医疗装备、智能驾驶等领域，突破300项关键共性技术，转化100项重大科技成果，高新技术企业达到1000家。加强协同创新，建立产学研内行碰撞、实时在线交流机制，推进45个产业创新联盟实体化运营。推广东北大学科技成果转化经验，提高科技成果本地转化率。设立并购基金，支持企业通过海外并购提高研发能力。

　　加快创新平台建设。推进沈阳材料科学国家研究中心、国家机器人创新中心、IC产业协同创新中心建设，打造“先进材料+智能制造”国家创新高地。引进深圳清华研究院、光启研究院、科大讯飞研究院、北京协同创新研究院等新型研发机构，支持企业建设院士工作站、工程技术研究中心和企业技术中心。强化沈阳科技条件平台功能，推动科研设备、人才服务、成果转化等资源开放共享。

　　优化创新创业生态。加快发展科技金融，创新政府性科技投资基金管理机制，大力引进天使、风投等股权投资基金。创建中国(沈阳)知识产权保护中心，实现专利“一站式”服务。支持专业化众创空间建设，布局科技孵化基地，营造创新文化氛围，深化国际科技合作，办好国际眼基因大会、材料科学高峰论坛等交流活动。

　　加强军民融合创新。统筹军转民、民参军和军民合作，出台军民融合政策措施，争创国家军民融合创新示范区。鼓励军工技术转移转化，推进中国航发燃气轮机示范应用等项目。推动军工龙头企业开放产业链，培育50户军工战略供应商。设立产业基金，壮大产业联盟，建设军民融合综合服务平台。

　　大力引进培养人才。坚持长期化、常态化、品牌化推进“三引三回”活动，搭好干事创业、投资兴业平台，带动各类人才加速回流。增强人才政策的针对性，引进高层次人才、紧缺人才和海外“高精尖”人才，培养中青年科技人才、高技能人才，落实好高校毕业生购房、创业补贴政策。深化人才管理体制改革，完善人才服务体系，增强人才的归属感，让更多人才在沈阳施展才华、创造财富、实现价值！

　　(六)不断深化改革扩大开放。深入推进全面创新改革试验，完善体制机制，进一步释放发展活力。

　　深化国资国企改革。落实市领导一对一、点对点工作机制，推动机床集团等12户重点企业改革取得新突破，“一企一策”改革在市属国有企业全面推开。完善法人治理结构，强化战略管控和风险防控，健全外部董事制度，实现外派监事会全覆盖。深化三项制度改革，扩大市场化选聘经理层范围，开展薪酬差异化试点。深化混合所有制改革，积极引进战略投资者，开展员工持股试点。充分发挥“3+1+N”国资运营平台作用，完善国资授权经营体制，加快由管资产向管资本转变，促进国有资本做强做优做大。下大力气解决历史遗留问题，启动厂办大集体改革，继续做好“三供一业”分离移交、“僵尸企业”处置工作。市属国有企业今年要整体扭亏为盈。

　　深化“多规合一”改革。推进底图叠合、数据融合、政策整合、管控协和，实现全域一张蓝图、全域“多规合一”。完善综合管理、空间协同、联合审批三大平台，实施项目救济机制，促进项目高效生成、快捷审批。积极推广应用“多规合一”成果，加快形成以规划为主导的经济社会管理体系。

　　深化财政体制改革。加强全口径预算编制管理，提高专项资金使用效率。创新政府资金使用方式，推进专项资金基金化运作，向中小企业、新兴产业倾斜。加强政府债务管控，严控总量、减少存量，严格界定政府投资范围，规范操作PPP项目、政府引导基金和政府购买服务，防范隐性债务风险。推进县乡财政体制改革，因地制宜实施“统分结合”“分灶吃饭”，增强乡镇自我发展和保障能力。

　　推进开发区改革创新。坚持把开发区(园区)作为项目建设的主阵地，完成行政区与开发区(园区)体制分离，复制推广“开发区+主题园区”“管委会+平台公司”模式。创新投融资方式，形成“开发-收益-再开发-再收益”的良性循环。完善招商引资政策，实施项目“满园扩园”“腾笼换鸟”行动，提高土地利用强度，各开发区(园区)要完成产业项目引进任务，力争地区生产总值、规模以上工业增加值和固定资产投资分别增长8%、9%和15%以上。

　　提高对外开放水平。创新自贸试验区体制机制，推进市场化开发建设运营。国际贸易“单一窗口”达到上海自贸区3.0升级版，深化“证照分离”改革，完善商事仲裁等涉外服务，实行准入前国民待遇和负面清单管理模式，促进服务业开放。确保综保区新B区封关运行，形成先进制造、再制造、融资租赁等产业形态。加快中德装备园建设，在技术标准、科技创新、双元培育等领域，加强中国制造2025与德国工业4.0对接。完成沈阳港集团重组，推进沈阳港项目建设。办好世界冬季城市市长会议等活动，复航沈阳至法兰克福航线，开通沈阳至洛杉矶航线。

　　推进沈阳经济区建设。联合制定一体化发展共同行动战略规划，不断增强连接聚合扩散能力。促进基础设施互联互通，推动桃仙机场二跑道、沈白高铁连通桃仙机场项目，完善城际路网体系。促进公共服务共建共享，加强教育合作，建设医疗联盟，共同开发旅游线路。促进产业协作配套，支持区域龙头企业开放产业链，协同建设生物医药等新兴产业园区。促进要素资源优化配置，共建科技创新服务平台和成果转化平台，推动金融市场、人才市场一体化。建立健全区域联动机制，加强生态环境共保共治。

　　深化京沈对口合作。积极承接北京疏解非首都功能，盯住央企、京企开展项目合作，抓好京东电商物流基地、联东主题产业园等重点项目建设。拓宽资源要素流动通道，聚焦资本、技术、人才，积极开展投资机构沈阳行、院士专家沈阳行，推动辽股交挂牌企业走进“新三板”，引入中关村元素，加快建设“一街、一园、一平台”。拓展市场合作领域，充分发挥龙头企业作用，健全两地商会、协会对接机制，广泛组织名优农产品、文化旅游产品北京行等活动。进一步抓好与江苏、厦门、深圳、延安等地区合作项目实施。

　　(七)提高城市规划建设管理水平。坚持精准规划、精致建设、精细管理，继续实施“治改提创”工程，不断提升城市品质。

　　强化规划引领和刚性管控。按照“一中心、多组团、串珠式”发展模式，融入海绵城市、紧凑城市、智慧城市理念，完成新一版总体规划编制改革试点，实现总体规划全域覆盖。完善专项规划和控详规划，加强城市设计，提升街区道路、公共设施、生态景观、地下管网等建设管理标准。加强存量土地攻坚，提高土地节约集约利用水平。

　　不断完善城市功能。启动浑南高品质公共服务新展区建设，推进盛京皇城等重点片区升级改造。完善综合交通体系，确保地铁9号线、10号线试运行，积极推动地铁3号线、6号线、2号线南延线以及有轨电车西部滨水线建设；启动浑南大道快速路、沈康高速连接线、四环棋盘山隧道工程，实施东一环、胜利南大街、沈辽路等快速路建设。加强交通节点治理，完成怒江北街延长线、陵园北街贯通工程，完善城市道路人行过街设施。增加城市慢行绿道50公里。完善城乡公用设施，加强电力、燃气应急储备设施建设，实施大伙房输水配套二期工程。推进海绵城市示范区建设，实施城市排水防涝、新民柳河防洪治理工程，完成南运河、铁西新城综合管廊项目，加大老旧管网改造力度，让城市的“面子”和“里子”同步升级！

　　加强城市精细化管理。抓好城市公共空间普查，制定精细化管理专项规范。深化城市管理执法体制改革，市、区县(市)全面建立大城管体制。打好拆违控违攻坚战，开展城乡接合部、背街小巷环境综合整治，清理盘活“停缓建”工程。完成扫保市场化改革，扫保机械化率达到85%以上。推进厕所革命，改造城区公厕172个。加强建筑立面综合整治和广告牌匾规范化管理，实施裸露地面软硬覆盖，提升城市亮化和景观水平。加快公交都市创建，新开调整公交线路30条，新增更新公交车900台，改造提升公交候车站3003处。新建盘活停车泊位20万个，探索共享停车新模式，努力缓解停车难问题。

　　持续改善生态环境。强化精准治霾、铁腕治霾，严格实施区域考核制度，深入开展挥发性有机物综合整治、燃煤替代、锅炉拆除、扬尘防控、秸秆禁烧和综合利用，空气质量优良天数比率提高1-2个百分点。强化水污染防治攻坚，开展建成区外围黑臭水体整治，建设东部污水处理厂，完成北部、西部、沈水湾、仙女河、满堂河污水处理厂提标改造，实施浑河、细河、蒲河、北沙河、左小河、八家子河超标断面治理，完成运河截污工程，实施百里环城水系连通和景观提升工程，加强水源地保护。提高垃圾处理能力，老虎冲、西部垃圾焚烧发电项目单体试运行，餐厨垃圾收运系统上线运行，开展垃圾强制分类管理试点。加快祝家污泥、大辛和老虎冲积存渗沥液安全处置。加强土壤污染防治，加大检测修复力度。实施退耕还林还湿还草还湖还生态工程，开展增绿补绿复绿造绿行动，植树造林15万亩。加强自然保护区监管，坚决制止和惩处破坏生态环境行为。加快建立碳排放权交易机制，力争年内实现交易。

　　(八)着力保障和改善民生。坚持尽力而为、量力而行，兜底线、织密网、建机制，让老百姓的获得感、幸福感和安全感在家门口不断升级。

　　提高就业和社会保障水平。着力解决就业结构性矛盾，促进高校毕业生、农民工多渠道就业，新增城镇就业9万人。实施全民参保计划，加强养老金扩面征缴和支出管理，有效防控支付风险，确保按时足额发放。提高城乡低保和农村特困供养标准，加大困境儿童、失独家庭、孤寡老人等特殊群体帮扶力度。推进居家和社区养老服务改革试点，提高养老院服务质量，新增养老床位2000张。推进住房租赁试点，培育20家专业化、机构化住房租赁企业，扩大租购同权事项。加强城中村、城边村综合治理，改造老旧小区120个、棚户区1.63万套，不断改善城乡居民居住条件。

　　优先发展教育事业。提高教育质量，加强公办幼儿园和中小学建设，普惠性幼儿园学位达到70%以上，进一步解决好中小学生课外负担重、“择校热”“大班额”等问题。加快普通高中教学改革，深化职业教育双元制改革，推动市属高校转型发展，支持驻沈高校建设一流大学和一流学科，进一步抓好特殊教育。建立责任管理体系，解决好婴幼儿照护和儿童早期教育服务问题，决不允许校园欺凌、伤童、虐童现象发生，守护好孩子们的美好童年！

　　繁荣发展社会事业。深化城市公立医院改革，加强医联体建设，扩大家庭医生签约服务覆盖率，推动医院检查结果互认，抓好市五院、传染病院、妇幼保健院、口腔医院浑南院区、中国医大浑南国际医院等项目建设。完善公共文化服务体系，办好2018中国国际合唱节。加强文物保护和文化遗产传承，启动建设北大营抗战遗址纪念馆等十大重点文化项目。统筹发展群众体育、竞技体育和体育产业，完善全民健身设施，制定实施三大球发展规划，办好沈阳国际马拉松、跃动沈阳国际运动汇等赛事活动。

　　加强和创新社会治理。巩固文明城市创建成果。扎实开展“三城联创”，确保进入国家卫生城市、国家食品安全示范城市行列。加强食品药品监管，让群众吃得更放心、用药更安全。创新社区治理，培育社会组织，深入推进共建共治共享。加强国防教育，增强全民国防意识。深化安全生产领域改革，提升防灾减灾救灾能力，坚决防止重特大安全事故发生。开展“融冰行动深化年”活动，依法及时解决群众合理诉求。加强立体化、信息化社会治安防控体系建设，不断夯实综合治理基层基础，严打黄赌毒、黑拐骗等违法犯罪活动，保持社会大局和谐安定。

　　(九)切实加强政府自身建设。实现新目标、完成新任务，必须建设高素质的干部队伍，不断提高政府公信力和执行力。

　　强化“四个意识”。扎实开展“不忘初心、牢记使命”主题教育，切实用习近平新时代中国特色社会主义思想武装头脑、指导实践、推动工作，始终在政治立场、政治方向、政治原则、政治道路上同以习近平同志为核心的党中央保持高度一致，坚决维护党中央权威和集中统一领导，坚决落实省委省政府和市委的各项要求部署以及系列政策性制度性文件。

　　坚持依法行政。自觉接受市人大及其常委会法律监督、工作监督和市政协民主监督，强化审计监督全覆盖，深化政务公开，主动接受社会监督、舆论监督。强化科学立法，坚持依法决策，健全公众参与和专家论证机制，完善集体讨论决定、终身追责及责任倒查机制。深化行政执法体制改革，全面推行重大执法决定法制审核制度，严格行政执法。完善公共法律服务体系，开展法治宣传教育，营造全社会尊法学法守法用法良好氛围。

　　加强纪律建设。始终把纪律和规矩挺在最前面，压紧压实全面从严治党主体责任，严肃党内政治生活，坚决破除宗派主义、圈子文化、码头文化，营造良好的政治生态。严格落实市委关于贯彻中央八项规定精神的实施细则，突出抓好形式主义、官僚主义新表现的整治。不断强化正风肃纪，严肃查处各类违规违纪行为，加大力度整治群众身边的腐败问题。

　　改进工作作风。认真落实市委关于大兴调查研究之风的意见，深入基层、深入群众、深入项目、深入企业，抓住突出矛盾，抓实基础工作，做好案头工作，严细实快、步步为营，以“马上办”“钉钉子”精神把各项工作办好办实、钉准钉牢。突破中梗阻，打通“最后一公里”，努力形成有目标、有路径、有措施、有时限、有结果的逻辑闭环，不断增强工作组织的穿透力。

　　各位代表，实现沈阳振兴发展，是人民群众的共同期盼，需要强烈的使命担当，需要付出极大的艰辛。只有干出来的精彩，没有等出来的辉煌。让我们更加紧密地团结在以习近平同志为核心的党中央周围，高举习近平新时代中国特色社会主义思想伟大旗帜，在市委的坚强领导下，撸起袖子干，甩开膀子干，脚踏实地干，齐心协力干，坚决完成全年各项目标任务，为决胜全面建成小康社会、实现沈阳老工业基地全面振兴而努力奋斗！
